# Intimate Partner Violence and Intimate Partner Homicide: Development of a Typology Based on Psychosocial Characteristics

**DOI:** 10.1177/08862605211021989

**Published:** 2021-06-02

**Authors:** Carolanne Vignola-Lévesque, Suzanne Léveillée

**Affiliations:** 1 University of Quebec at Trois-Rivières, Quebec, Canada

**Keywords:** intimate partner violence, intimate partner homicide, typology, alexithymia

## Abstract

Intimate partner violence (IPV) remains an important and alarming global issue. Studies have put forth different profiles of perpetrators of IPV according to the severity of the violence and the presence of psychopathology. The objective of this study was to develop a typology of perpetrators of IPV and intimate partner homicide (IPH) according to their criminological, situational, and psychological characteristics, such as alexithymia. Alexithymia is when a person has difficulty identifying and describing emotions and in distinguishing feelings from bodily sensations of emotional arousal. Data were collected from 67 male perpetrators of IPV and/or homicide. Cluster analyses suggest four profiles: the homicial abandoned partner (19.4%), the generally angry/aggressive partner (23.9%), the controlling violent partner (34.3%), and the unstable dependent partner (22.4%). Comparative analyses show that the majority of the homicidal abandoned partners had committed IPH, had experienced the breakup of a relationship, and had a history of self-destructive behaviors; the generally angry/aggressive partners were perpetrators of IPV without homicide with a criminal history and who were alexithymic; the controlling violent partners had a criminal lifestyle and committed IPH; and the unstable dependent partners had committed IPV without homicide, were alexithymic, but had no criminal history. Establish a better understanding of the psychological issues within each profile of perpetrators of violence within the couple can help promote the prevention of IPV and can help devise interventions for these individuals.

## Introduction

Intimate partner violence (IPV) is a major problem worldwide. More than 403,201 people were victims of a violent crime in 2017, 30% of whom were abused by an intimate partner (Beattie et al., 2018). In 2018, 99,452 cases of domestic violence were reported to police in Canada (Conroy et al., 2019). Over half of intimate partner victims (56%) sustained physical injury, while 5% of victims report sexual violence. Major injuries and death resulted in 2% of victims. Intimate partner homicide (IPH) is a subtype of IPV. In 2019, police reported 678 homicides in Canada. Half of these homicides committed involved a current or former intimate relationship, including spouses. According to police-reported statistics, women are overrepresented as victims of IPV, accounting for almost 8 in 10 victims (79%) (Conroy et al., 2019). In 2015, Quebec’s police services recorded 36 attempted murders within an intimate partner context as well as 11 IPHs (Gouvernement du Québec, 2017). Of these victims, 78% were women. In Canada, 51 IPHs were committed in 2017, representing 11.6% of all homicides committed across the country (Burczycka et al., 2018).

Recent studies investigating the psychosocial issues of perpetrators of IPV or IPH show that there is no unique profile of perpetrators of these types of violence. In fact, each subgroup of perpetrators presents specific characteristics (Adams, 2007; Dutton, 2007; Elisha et al., 2010; Khoshnood & Fritz, 2017). However, few of the typologies identified in these studies include psychological variables associated with emotional management, such as alexithymia. Alexithymia is a personality construct characterized by difficulties in recognizing and distinguishing different emotions and bodily sensations, difficulties in expressing emotions, a lack of imagination or fantasy life, and thoughts focused on external rather than internal experience (Sifneos, 1973; Taylor et al., 1999). Yet, certain psychological vulnerabilities, such as a difficulty in identifying and expressing one’s emotions, could make one more likely to adopt violent behavior and commit a homicide (Hornsveld & Kraaimaat, 2012; Léveillée, 2001). Given that certain psychological characteristics, combined with external factors such as the context of the violent behaviors, a history of violence, and past suicidal behaviors, are associated with violence within the couple (Di Piazza et al., 2017), it is particularly important to describe the psychosocial characteristics of perpetrators of IPV and IPH in order to explore the link between these characteristics and the type of violence committed. The advancement of knowledge in this area could enable practitioners working with perpetrators of IPV, or who work in settings with individuals who are likely to exert this type of violence, to plan clinical strategies adapted to their specificities and difficulties, by focusing both on internal (psychological characteristics) and external factors (context of violence, history of violence, history of self-destructive behaviors, etc.). Looking at different profiles of perpetrators of IPV may be a first step towards treatments adapted specifically to different profiles or even to prevent the IPH through interventions. These prevention and intervention strategies must be adapted to the psychological challenges experienced by these men, with the aim of promoting the rehabilitation of victims of IPV.

## Intimate Partner Violence

According to Quebec’s government action plan for IPV 2018–2023, IPV is characterized by a “series of repetitive acts, which generally occur in an upward trend.” Experts describe this progression as an “escalation of violence” in successive phases, which are marked by increased tension and aggression. IPV does not result from a loss of control but rather represents a chosen means by the aggressor to dominate the other person and assert their power over them (Gouvernement du Québec, 2018). These violent behaviors, which are committed by an intimate partner or ex-partner, can be manifested in several forms: physical, sexual, psychological, verbal, and economic violence. Physical violence includes actions that cause physical injury. Sexual violence encompasses all forms of violence, physical and psychological, that violate a person’s sexual integrity. Psychological violence is the devaluation of the other through contemptuous remarks, coercion, and isolation, while verbal violence involves creating a feeling of terror through insults and threats. Finally, economic violence aims to inflict financial consequences on the victim through deprivation of monetary and material resources (Gouvernement du Québec, 2018). The most severe form of IPV is IPH and includes homicides in which the alleged perpetrator is the partner, whether they are married, separated or divorced, common-law partner (current or former), or a close friend of the victim (Beaupré, 2015). Given its multifactorial origin and the fact that this type of violence occurs within a context of intimacy, the prevention of IPH remains complex. IPH can be understood as the expression of a feeling of possessiveness as well as a refusal to lose control over one’s partner (Drouin et al., 2012). This type of violent act is still not sufficiently taken into consideration in prevention and intervention programs, as it is often considered to be the result of increasingly severe and intense IPV. However, like the modus operandi, the profile of perpetrators of IPH differs from those of other types of IPV and homicide. Additionally, some perpetrators of IPH had no known history of violence prior to the homicide. More empirical support is needed to better understand the links between the psychological and social characteristics and the different types of violence committed within the couple. However, few studies have examined the combination of criminological, situational, and psychological factors among perpetrators of IPV, including IPH.

### Risk Factors of Intimate Partner Violence

Several reviews of the literature on factors associated with IPV (Capaldi et al., 2012; Schumacher et al., 2001) and IPH (Campbell et al., 2007) have been published in order to gain a better understanding of the characteristics that increase the probability of an individual committing violence within the couple.^1^ These factors focus mainly on sociodemographic variables, situational variables, and the characteristics of the violence committed (Aldridge & Browne, 2003; Capaldi et al., 2012). For example, lower age, unemployment, and low education have been identified as risk factors of IPV (Capaldi et al., 2012), while studies reported that the majority of IPH perpetrators tend to be older, employed, and have medium socio-economic status (Dobash et al., 2009).

Life circumstances and contextual factors are likely to influence the manifestations of violent behaviors in the context of an intimate relationship (Yoshihama & Bybee, 2011). Among the different life circumstances that may occur, the breakup of a relationship appears to be one of the most frequent triggers of IPV (Léveillée et al., 2017; Notredame et al., 2019; Sinha, 2013). In 2015, 32.8% of offenses committed in the context of a relationship that was reported to the Quebec’s police were committed by an ex-partner (Ministère de la Sécurité publique, 2017). In fact, the breakup of a relationship represents a major risk factor associated with IPH (Abrunhosa et al., 2020). The period immediately preceding or following the breakup is when the risk of homicide is at its highest (Campbell et al., 2007; Léveillée & Lefebvre, 2011). A breakup leads to emotional distress and a feeling of rejection, and thus constitutes a period of considerable vulnerability for these men who already have psychological difficulties (Drouin et al., 2012; Léveillée et al., 2017). Violent behaviors are therefore used to maintain control over the partner (Kelly & Johnson, 2008).

Certain individual characteristics are associated with the risk of violence in the context of a marital separation including the presence of a criminal history (Piquero et al., 2013). According to Piquero et al. (2006), the majority of perpetrators of IPV have committed other non-violent crimes. Other studies have revealed the presence of a known criminal history of IPV, a history of physical assault toward another person, and a history of offenses related to drug or alcohol use (Lévesque et al., 2009). The presence of a criminal history increases the risk of perpetrators of IPV reoffending, which could result in a new offense toward their partner or a breach of the terms of their probation (Kingsnorth, 2006). The history of violence of perpetrators of IPH is essentially present within the intimate relationship (Campbell et al., 2007). Among the IPHs in Canada between 2001 and 2011, 78% of cases indicate the presence of a history of violence known to the police between the victim and the perpetrator (Sinha, 2013). Stalking behavior, the availability of handguns, and alcohol or drug abuse are also recognized as risk factors for IPH (Aldridge & Browne, 2003; Campbell et al., 2007). Although identifying risk factors among IPV and IPH perpetrators helps better prevent the risk of violence, few studies have focused on psychological factors associated with these types of violence.

### Psychological Characteristics of Perpetrators of Intimate Partner Violence

In many cases, the violent behavior does not appear to be committed exclusively towards others. Indeed, a high proportion of perpetrators of IPV have a history of violence towards themselves (Devries et al., 2013; Léveillée et al., 2009; Wolford-Clevenger et al., 2015). Studies evaluating the link between IPV behaviors and the risk of suicide have shown that this relationship is particularly strong in male perpetrators of severe violence who are involved in a legal process (Conner et al., 2002; Léveillée et al., 2009). The presence of interpersonal and legal problems are predictors of suicide attempts, beyond the presence of personality disorders (Yen et al., 2005). Suicidal behaviors, such as suicide attempts or a completed suicide following a homicide, are also seen among perpetrators of IPH (Léveillée et al., 2017). In most cases, the suicide or attempted suicide occurs immediately following the homicide (Aston & Bunge, 2005). Between 2001 and 2011, 54% of homicide-suicide cases involved men who killed their ex-partner (Brennan & Boyce, 2013).

The adoption of destructive behaviors, whether they are committed towards others or towards oneself, may reflect the presence of a deficit in emotional management (Hornsveld & Kraaimaat, 2012; Porcelli & Mihura, 2010). Indeed, impulsivity, depressive affects, and difficulty in managing anger are characteristic of perpetrators of IPV (Di Piazza et al., 2017; Léveillée et al., 2009; Shorey et al., 2011). However, studies show that difficulty identifying and communicating emotions is associated with depression, the adoption of impulsive behaviors, and relationship difficulties (Grynberg et al., 2010; Vanheule et al., 2010). This deficit is called alexithymia and is characterized by (a) an inability to identify and verbally express one’s emotions and feelings; (b) a limited fantasy life; (c) pragmatic thinking accompanied by a very descriptive mode of expression; and (d) recourse to action to avoid conflict or the expression of emotions (Corcos & Speranza, 2003). Individuals who have difficulty understanding or verbalizing their emotional experiences are more at risk of engaging in aggressive behaviors as a way to regulate their emotions (Cohn et al., 2010). Some studies show the presence of alexithymia in more than half of perpetrators of IPV (Di Piazza et al., 2017; Léveillée & Vignola-Lévesque, 2019).

Although understanding the characteristics of IPV and IPH perpetrators and the risk factors within these relationships will aid in the recognition of risk of lethality, studies have shown that the joint presence of certain risk factors can significantly increase the risk of committing violent acts within the couple (Dutton, 2007). Based on this finding, researchers have identified different subgroups of perpetrators of IPV, each with distinct characteristics (e.g., Adams, 2007; Johnson, 2008).

### Typologies of Perpetrators of Intimate Partner Violence

Given that no single profile of perpetrators of IPV exists, some researchers have developed typologies of men who perpetrate violent behaviors towards their partner (de Mijolla-Mellor, 2017; Dutton, 2007; Elisha et al., 2010; Kivisto, 2015). Dutton’s typology (2007) is particularly useful and is based on clinical observations and results obtained from the *Millon Clinical Multiaxial Inventory II* (MCMI-II).^2^ Dutton suggests that there are three subgroups of men, which include those who exhibit impulsiveness, those who act out for utilitarian purposes, and those who tend to avoid conflict and anger. According to Dutton (2007), those who tend to avoid conflict are more at risk of committing IPH.

Male perpetrators of IPH also have distinct profiles. Adams (2007) assessed the characteristics of different clinical cases of men who committed homicide or attempted IPH. Based on the men’s behaviors, attitudes, and relational history, this researcher identified five types of men who perpetrate IPH: the jealous partner, the substance abuser, the suicidal partner, the partner who is motivated by pecuniary benefits, and the partner with a criminal history. Elisha et al. (2010) identified male perpetrators according to three subgroups. The first subgroup includes individuals with a stable lifestyle and no history of IPV. Perpetrators from the latter tend to commit homicide to punish their partner for breaking the family structure. The second subgroup includes individuals with borderline personality structure as well as a dependency on their partner. A breakup or the mere mention of a breakup by the partner is a trigger of this type of homicide. The third subgroup comprises violent and emotionally unstable individuals. These men, who have a criminal lifestyle, commit homicide in response to their desire to gain control over their partner. More recently, Kivisto (2015) proposed, based on a review of the literature, a typology of perpetrators of IPV which includes four profiles. The first profile comprises perpetrators of IPH with psychotic or depressive disorder. These individuals are less likely to have committed IPV prior to the homicide but are more likely to have also killed other members of their family at the time of the crime. The second profile includes impulsive individuals who present with borderline personality disorder,^3^ jealousy, a fear of abandonment, and problematic alcohol or drug use. The third profile includes perpetrators of chronic IPV. These individuals have antisocial or narcissistic personality disorder and have generally committed other violent crimes prior to the homicide. Finally, the fourth profile refers to overcontrolled/catathymic individuals with a dependent or schizoid personality disorder and who experience envy towards others.

The findings from studies on perpetrators of IPV raise the possibility that certain personal, situational, and criminological variables may have an influence on the different types of violent behaviors that occur within the couple. Although some researchers have looked into the presence of a mental health disorder among these men (Dutton, 2007; Kivisto, 2015), no study, to the best of our knowledge, has identified profiles of perpetrators of different types of IPV by including variables that may affect psychological functioning and emotional management, such as alexithymia. The assessment of alexithymia would inform practitioners and professionals regarding the recommended intervention targets for perpetrators of IPV. Investigating profiles by combining situational, criminological, and psychological characteristics could potentially provide a more detailed portrait of the subgroups of men who perpetrate violent behaviors in their intimate relationships and develop a typology including issues of emotional regulation in these individuals.

### Objectives

The first objective of this study is to identify the presence of profiles of male perpetrators of IPV and/or IPH and to describe the constitution of these profiles according to criminological, situational, and psychological characteristics. The second objective is to verify, in the event that profiles of individuals are indeed identified, the participants’ distinct and similar characteristics according to the profile they belong to.

## Method

### Participants

The sample for the present study comprises 67 male perpetrators of IPV, including 45 perpetrators of IPV who did not commit IPH (mean age = 41.36 years, *SD* = 9.04) and 22 perpetrators of IPH (mean age = 52.24 years, *SD* = 12.54). At the time of the homicide, these men were on average 39.78 years old (*SD* = 11.37). The perpetrators of IPV without homicide were recruited from a support center for individuals of violent and controlling behaviors towards their partner. The perpetrators of IPH were recruited from detention centers from the Correctional Service of Canada, where they are serving a sentence of more than two years for the homicide of their partner.^4^ Demographic characteristics are presented in Table 1. More than half of the IPV perpetrators (*n* = 25; 55.6%) had criminal history. Half of IPV perpetrators were voluntary patients at the support center (*n* = 21; 49,7%), while the other half were court-ordered (*n* = 24; 53,3%). Most IPH perpetrators (*n* = 14; 63.6%) had a history of IPV. The information reported concerns the characteristics of the men at the time of their offense.

**Table 1.** Demographic Characteristics of Intimate Partner Violence Perpetrators.

Variables^a^	*n*	%
Age	*M* = 41.33 *SD* = 9.67	
Marital status		
Common-law	22	32.8
Married	18	26.9
Divorced/separated	27	40.3
Employed	49	73.1
Education		
Elementary or secondary	47	70.1
Vocational studies	13	19.4
College or university	7	10.4
Children	53	79.1

*Notes*. *SD* = Standard deviation.

^a^These variables are relevant to the time of the incident.

### Measures

In the context of the present study, the variables were collected during semi-structured interviews carried out with the participants. The variables used, which were chosen based on the literature on characteristics of perpetrators of IPV and IPH include criminological characteristics (the type of IPV committed and the presence of a known criminal history—excluding a criminal history of IPV), situational characteristics (the presence of the breakup of a relationship) and psychological characteristics (the presence of one or several suicide attempts—excluding suicide attempts directly as a result of the homicide—and the presence of alexithymia).

Alexithymia was assessed using the *Toronto Alexithymia Scale* (TAS–20). The TAS–20 (Bagby et al., 1994) is a scale used to assess the presence of alexithymia and its three clinical dimensions:

1. difficulty in identifying one’s emotions and those of others,
2. difficulty in describing one’s emotions, and
3. operative or outward-oriented thinking.

The participant indicates their level of agreement or disagreement for each of the 20 statements on a scale from 1 (complete disagreement) to 5 (complete agreement). The total score varies between 20 and 100. A score below 45 indicates that the individual is non-alexithymic, a score between 45 and 56 indicates that the individual is at the threshold of alexithymia (sub-alexithymia), and a score greater than or equal to 56 indicates that the individual is considered alexithymic. Alexithymia cutoff scores refer to the presence of certain characteristics of alexithymia, but which are insufficient to meet the presence of alexithymia (Luminet et al., 2003). This questionnaire has an internal consistency (Cronbach’s alpha of .79; Loas et al., 1995) and a test-retest stability (.77; Bagby et al., 1994) that are deemed satisfactory.

### Procedure

This study is part of a larger project focusing on the psychological changes and psychological issues of perpetrators of IPV (Léveillée et al., 2013). Recruitment was carried out through staff from each organization.^5^ These individuals suggested this study to potential participants and obtained their written consent for an individual meeting with a researcher. Then, this same researcher presented the research consent form and began interviewing participants if they agreed to participate. Data collection was carried out through semi-structured interviews. Given that IPV is a sensitive and complex subject, this type of interview offers the possibility to develop a deeper understanding of the participants (Savoie-Zajc, 2009). These semi-structured interviews provided us with access to sociodemographic and criminological information, as well as the context of the violent behaviors committed.^6^ Next, the alexithymia questionnaire (*TAS–20*) was administered to participants. This research project was approved by the ethics committee of the psychology department of the University of Quebec at Trois-Rivières (CER–07–121–07–10).

### Data Analysis

Data were processed using the SPSS 26 (2018) software (IBM Corp. Released., 2018). Descriptive analyses were first carried out in order to identify the sociodemographic characteristics of the participants. Then, a classification analysis was performed in order to group the participants according to a selection of relevant variables that had been associated with IPV and IPH in the literature: relationship breakup, criminal history, suicide attempt, and alexithymia. These variables were selected since they provide information on psychosocial and criminological characteristics of perpetrators of IPV and IPH. This study is exploratory. Classification analysis serves as a useful method for exploring and examining heterogeneity of a population of intimate partner perpetrators and for identifying groups of individuals who share the same characteristics. Participants in a specific subgroup share similar characteristics but differ from participants in other subgroups (Sarstedt & Mooi, 2014). In the present study, classification analysis was used to identify groups of male perpetrators of IPV based on the type of violence committed, the context of the violence, the presence of a criminal history, and psychological characteristics. Prior to running the algorithm, tetrachoric correlations were used to verify and control the collinearity and ensure that some variables don’t get a higher weight than others in the cluster analysis. The correlations results showed the absence of collinearity, with correlation coefficients >.90, as suggested by Sarstedt and Mooi (2014). Given the presence of categorical variables, the *Cluster Two-Step* analysis (Chiu et al., 2001) based on the *Bayesian Information Criterion* (BIC) was selected to group participants. The number of clusters was determined using the coefficient of agglomeration and the dendrogram. Next, Chi-square analyses were performed to check whether the characteristics of perpetrators of IPV differed significantly from one profile to another. In cases where Chi-square analyses indicated the presence of significant differences between profiles, a posteriori analyses using the Bonferroni–Holm correction (1979) were conducted in order to identify the differences that were most likely to be significant.

## Results

**Figure 1.**
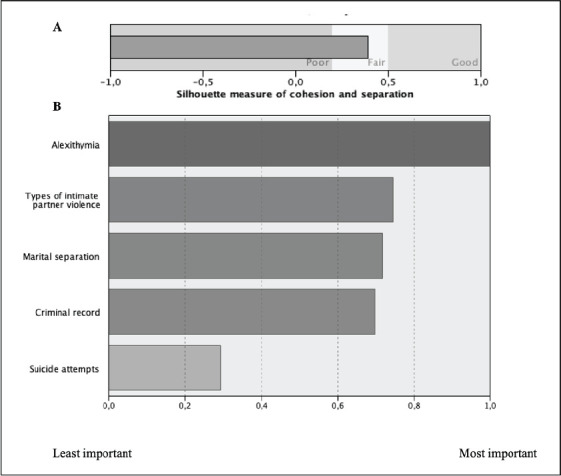
Results from the cluster analyses. (A) Silhouette measure of cohesion and separation. (B) Relative importance of variables in the creation of profiles.

The *Two-Step* classification analysis identified four distinct profiles (Table 2). The size ratio is 1.77. The quality of cohesion and separation is sufficient, with a silhouette coefficient of .4 (Figure 1A). The silhouette coefficients of the two-and three-profile solutions were .3, showing a lower quality of data classification for these models, which explains the rejection of these solutions. The index of relative importance of the variables in the creation of the profiles shows that the most determining variable for the classification of participants is alexithymia (Figure 1B).

**Table 2.** Distribution of Clusters and Automatic Creation of Clusters.

Group	Number of Participants/Group	Group Size (%)	BIC	BIC Change^a^	Distance Measurement Report^b^
1	13	19.4	508.05	–	–
2	16	23.9	436.32	–71.73	1.27
3	23	34.3	384.88	–51.44	1.25
4	15	22.4	348.72	–36.17	1.46
Total	67	100.0	–	–	–

*Notes*. ^a^The changes correspond to the previous number of clusters in the table.

^b^Distance measurement reports are based on the current number of clusters, compared to the previous number of clusters.

Each profile has been named according to the main psychosocial issues that characterize the functioning and psychosocial issues of perpetrators of IPV:

1. the homicidal abandoned partner;
2. the generally angry/aggressive partner;
3. the controlling violent partner, and
4. the unstable dependent partner (Table 3).^7^

Profile 1 (the controlling abandoned partner; *n* = 13) comprises only perpetrators of IPH. All of these men (100%) experienced a relationship breakup and had attempted suicide at least once in their lifetime. Among the individuals grouped in this profile, 38.5% had a known criminal history and 69.2% presented with sub-alexithymic functioning. Profile 2 (the generally angry/aggressive partner; *n* = 16) consists only of perpetrators of IPV who did not commit IPH. All individuals in this profile (100%) had a known criminal history. Of these, 50.0% experienced a relationship breakup, 56.3% had attempted suicide at least once, and 93.8% were alexithymic. Profile 3 (the controlling violent partner; *n* = 23) comprises 30.4% of the perpetrators of IPH and 69.6% of the perpetrators of IPV. Among the individuals included in this profile, 17.4% had experienced the breakup of a relationship, 69.6% had a known criminal history, and 34.8% had attempted suicide at least once. 87.0% of these individuals exhibited sub-alexithymic functioning, while 13.0% were non-alexithymic. Profile 4 (the unstable dependent partner; *n* = 15) comprises 13.3% of perpetrators of IPH and 86.7% of perpetrators of IPV who did not commit IPH. None of them had experienced a relationship breakup and none had a known criminal history. All were alexithymic and 40.0% had attempted suicide at least once.

**Table 3.** Characteristics of Perpetrators of Intimate Partner Violence According to Their Profile.

Characteristics	Profile 1 The homicidal Abandoned Partner *n* = 13 % (*n*)	Profile 2 The Generally Angry/Aggressive Partner *n* = 16 % (*n*)	Profile 3 The Controlling Violent Partner *n* = 23 % (*n*)	Profile 4 The Unstable Dependent Partner *n* = 15 % (*n*)
Type of violence committed				
Intimate partner violence	0	100.0 (16)	30.4 (7)	86.7 (13)
Intimate partner homicide	100.0 (13)	0	69.6 (16)	13.3 (2)
Relationship breakup				
Yes	100.0 (13)	50.0 (8)	17.4 (4)	0
No	0	50.0 (8)	82.6 (19)	100.0 (15)
Criminal history				
Yes	38.5 (5)	100.0 (16)	69.6 (16)	0
No	61.5 (8)	0	30.4 (7)	100.0 (15)
Suicide attempt				
Yes	100.0 (13)	56.3 (9)	34.8 (8)	40.0 (6)
No	0	43.8 (7)	65.2 (15)	60.0 (9)
Alexithymia				
Non-alexithymic	15.4 (2)	6.3 (1)	13.0 (3)	0
Sub-alexithymic	69.2 (9)	0	87.0 (20)	0
Alexithymic	15.4 (2)	93.8 (15)	0	100 (15)

Chi-square analyses show a statistically significant difference between the profiles with respect to the presence of a relationship breakup, *X*^2^(3) = 35.772, *p* < .001, with a large effect size, Cramer’s V = .731, criminal history, *X*^2^(3) = 34.863, *p* < .001, Cramer’s V = .721, and the presence of at least one suicide attempt, *X*^2^(3) = 15.695, *p* = .001, Cramer’s V = .484. There is also a significant difference between the profiles regarding the type of IPV committed, *X*^2^(3) = 37.060, *p* < .001, Cramer’s V = .744, and the presence of alexithymia, *X*^2^(6) = 57.572, *p* < .001, Cramer’s V = .655. A posteriori analyses using the Bonferroni–Holm correction indicate that the individuals from profile 1 are significantly more to have experienced the breakup of a relationship (*p* < .001), while individuals from profile 4 are significantly less to have experienced the breakup of a relationship (*p* = .006) compared to the other profiles. Individuals from profile 2 are significantly more to have a known criminal history (*p* < .001), while individuals from profile 4 are those who present it the least (*p* < .001). Next, individuals from profile 1 are significantly more to have attempted suicide (*p* = .002). Profile 1 includes significantly more perpetrators of IPH and fewer perpetrators of IPV without homicide (*p* < .001), while profile 2 includes more perpetrators of IPV (*p* = .010) compared to other profiles. Lastly, the individuals from profiles 2 (*p* = .006) and 4 (*p* = .001) are significantly more to have alexithymia compared to individuals from the other groups. Individuals from profile 3 are more to present sub-alexithymic functioning (*p* < .001).

## Discussion

The objective of this study was to verify the existence of profiles of male perpetrators of IPV or IPH and to explore the distinct and similar characteristics between these groups. Four profiles were highlighted. Our findings show that the individuals from each profile have distinct situational, criminological, and psychological characteristics.

The first profile (the homicidal abandoned partner) includes individuals who perpetrate IPH, who have experienced the breakup of a relationship shortly before the homicide, and who have made at least one suicide attempt. Only a few of these individuals have a known criminal history. Most of these men have a sub-alexithymic functioning, that is, they only present a few characteristics of alexithymia. Profile 1 can be compared to the “*Abandoned obsessive lover*” subgroup identified by Elisha et al. (2010), both of which indicate that some men kill their former intimate partner after the partner has decided to end the relationship. Dutton (2007) also identified a subgroup of “overcontrolled” perpetrators of IPV who exhibit perfectionistic tendencies and conflict avoidance. It is possible to hypothesize that the loss of a partner’s love grows intolerable and causes an intense emotional charge that is difficult to formulate psychologically. The homicide can represent an attempt to gain ultimate control over the ex-partner. More studies are needed to confirm this explanation. The presence of sub-alexithymic functioning in these men shows that they can, under certain difficult circumstances, have difficulty identifying, working out, and verbalizing their emotions (Léveillée & Vignola-Lévesque, 2019). The presence of a history of suicide attempt(s) also confirms the psychological distress of these men and their difficulty in dealing with their emotions (Léveillée et al., 2017). Moreover, the results pertaining to the criminal history among the men from this profile are coherent with those from Abrunhosa et al. (2020), which revealed the presence of a criminal history in 38% of their sample of perpetrators of IPH. For these men, the difficulty in managing their aggressiveness and their need for control seems to be manifested mainly within the sphere of the couple’s relationship.

The second profile (the generally angry/aggressive partner) includes individuals who perpetrate IPV without having committed IPH but who have a criminal history. Half of the men from this sample had experienced a relationship breakup and had made at least one suicide attempt in their lifetime. Most of these men were alexithymic. These results are similar to those from several other studies (Cunha & Gonçalves, 2016; Dutton, 2007; Léveillée & Lefebvre, 2008; Piquero et al., 2013) and support the hypothesis that several perpetrators of IPV have difficulty identifying, verbalizing, and dealing with their emotions and aggressiveness, which manifest through violent behaviors both towards their partner and outside of the relationship. According to some studies (Deslauriers & Cusson, 2014; Dutton, 2007), a subgroup of perpetrators of IPV engage in serious (both in frequency and severity) violent behaviors, feel little remorse and little empathy towards others, and are violent in contexts outside of the sphere of the intimate relationship. Additionally, some perpetrators of IPV exhibit a tendency towards manipulation and a lack of empathy in interpersonal relationships, which can lead to more violent behaviors towards their partner (Cunha & Gonçalves, 2013; Huss & Langhinrichsen-Rohling, 2006). Violence and control are used as ways to emotionally regulate a heavy aggressive charge that cannot be verbalized.

A third profile (the controlling violent partner) consists mainly of individuals who perpetrate IPH, who have a criminal history, and who have not experienced a relationship breakup. Few of them had attempted suicide. Most of these men presented a sub-alexithymic functioning. This subgroup has also been identified in other studies (e.g., Elisha et al., 2010) and includes men who are unstable, violent, and who have a criminal lifestyle. The issues related to the desire to control their partner, as well as their emotional instability, are typical characteristics of these men (Dutton, 2007). One hypothesis that can explain this result is that despite they are better able to identify and process their emotions in some contexts, it appears that, at the time of the crime, these men experienced an intolerable emotional overload that they were unable to handle, and which eventually led to the homicide of their partner. This hypothesis remains to be confirmed.

Finally, a fourth profile of individuals (the unstable dependent partner) mainly includes perpetrators of IPV who do not have a criminal history and who have not experienced a relationship breakup. Less than half of these men had attempted suicide. All of these men were alexithymic. This profile is comparable to the *dysphoric-borderline* subgroup identified by Holtzworth-Munroe and Stuart (1994), Johnson’s (2008) “intimate dependent terrorist,” and to the “cyclical” subgroup identified by Dutton (2007). These men engage in low-to-moderate violence that rarely, if ever, spills outside of the couple. These men have difficulty coping and verbalizing their anger, anxiety, and depressive affects, which explains the presence of alexithymia. The use of violence in the relationship is an inadequate problem-solving strategy that allows them to avoid experiencing abandonment (Di Piazza et al., 2017; Norlander & Eckhardt, 2005).

The second objective of this study was to verify the presence of significant differences between the profiles of individuals in terms of the type of violence committed, the presence of a relationship breakup, the presence of a criminal history, of suicide attempt(s), and alexithymia. Alexithymia appears to be a key variable in the understanding of IPV, since it characterizes the psychological functioning of all perpetrators of IPV, including perpetrators of IPH. However, our results show particularities concerning this variable depending on the subgroups. Indeed, the perpetrators of IPV who have not committed a homicide (the “generally angry/aggressive partners” and the “unstable dependent partners”) are largely alexithymic, while perpetrators of IPH (the “homicidal abandoned partners” and the “controlling violent partners” perpetrators) present sub-alexithymic functioning. Léveillée & Vignola-Lévesque (2019) also show a higher percentage of perpetrators of IPV who are alexithymic compared to perpetrators of IPH. Although perpetrators of IPV exhibit controlling and dominating behaviors in intimate relationships, there appear to be particularities within each of these groups. Perpetrators of IPV who have not committed homicide have a greater difficulty identifying and verbalizing their emotional experiences, leading to the recurrence of violence in the relationship as a means of emotional regulation. Perpetrators of IPH appear to exert excessive control over their emotional reactions within the relationship, including trying to inhibit their anger and aggression. The breakup of the relationship, which represents a triggering event, risks greatly disrupting this excessive control and could lead to an emotional overflow within the individual. Several studies (Jouanne, 2006; Kowal et al., 2020) show that alexithymia reflects either an emotional deficit (alexithymia-state) or a stable personality trait (alexithymia-trait). Thus, certain perpetrators of IPV present primary alexithymia, which is integrated into their personality structure. Alexithymia as a stable trait increases vulnerability to stress and emotionally charged situations (Zimmermann et al., 2008). Violent acts allow the individual to reduce the bodily tensions associated with difficult emotions. The hypothesis of secondary alexithymia is considered for perpetrators of IPV. Secondary alexithymia refers to regressive psychological functioning, which allows affects to be blocked when faced with acutely stressful or traumatic situations that the individual is unable to cope with psychologically (Léveillée & Vignola-Lévesque, 2019). Marital separation can increase the intensity of the individual’s controlling behaviors, and could even lead to the death of the other. The violence exerted by perpetrators of homicide represents an attempt to have ultimate control over their (ex-)partner in a situation of vulnerability and intense stress. In contrast to the other subgroups, the majority of the “homicidal abandoned partners” and the “angry/aggressive criminal partners” have made at least one suicide attempt in their lifetime. These men have a strong propensity to turn their aggressiveness towards themselves. Self-destruction appears to be an important issue for the men from these subgroups, as they are associated with emotional distress that is acted out without being mentalized (Léveillée et al., 2017; Wolford-Clevenger & Smith, 2017).

Given the distinctions between the profiles identified in the present study, the identification of the emotional deficits of these men should be more explicitly measure. Assessing the psychological characteristics of these men could promote interventions that are tailored to their needs. Additionally, our study highlights the heterogeneity of the psychological profiles of perpetrators of IPV and indicates that some men are better able to identify and verbalize their emotional experiences than others. These emotional abilities certainly influence the therapeutic process and the therapeutic choices when working with these individuals (Léveillée et al., 2020). In addition, our results show the relevance of gaining a better understanding of the psychological characteristics of perpetrators of IPV. However, according to the literature, too few studies have explored alexithymia within various groups of perpetrators of IPV and IPH. Although the profile analysis represents an innovative contribution and involves semi-structured interviews with participants, these profiles should be confirmed with a larger sample. In fact, one of the study’s limitations is the fact that one of the profiles only consists of 13 participants, which inevitably influences the quality of the classification process. Using a self-report measure to assess psychological issues may lead to social desirability response bias or be biased by their difficulty to describe their emotional experiences. Additionally, the number of variables evaluated in this study is limited. Although this study is one of the only studies that has evaluated the criminological, situational, and psychological variables in perpetrators of IPV, other factors are likely to explain an individual’s affiliation to a particular perpetrator profile. Indeed, there are several forms of IPV that can vary in severity and intensity (not only violence or homicide). This heterogeneity in individual and violence-related characteristics could be considered in future studies. Our sample includes individuals who are starting or are currently in treatment for their violence, which could have had an impact on their ability to identify and verbalize their emotions. However, our results show that the majority of IPV perpetrators are alexithymic, although they are taking part in treatment. Further studies should include men at different stages of change regarding their violent behavior. Our study shows that alexithymia is an important variable for the understanding of IPV, but it cannot alone explain the adoption of violent behaviors within the couple. Spencer and Stith’s (2018) meta-analysis, namely, reports the presence of jealousy and personality disorders among perpetrators of IPV. Studies could include other psychological characteristics associated with emotional management, such as impulsivity, depressive affects, and mentalization skills, in order to understand the internal issues associated with perpetrator profiles.

## Conclusion

This study helped identify four profiles of male perpetrators of IPV based on criminological, situational, and psychological variables. The results show that some factors are associated with IPH, while others are more characteristic of perpetrators of IPV without homicide. These observations are of clinical interest for practitioners working with perpetrators of IPV or for practitioners who come into contact with these individuals during their practice. Psychological characteristics, including alexithymia, are key variables that can help better understand one’s use of violent behaviors within the couple. Our results encourage practitioners to focus on these individuals’ abilities to manage their emotions and on their mentalization skills. Research on the psychological processes is essential in order to develop prevention and intervention plans that are adapted to the needs, vulnerabilities, and strengths of men who perpetrate IPV.

## Notes

1. Actuarial tools have been designed to assess the risk of domestic violence, such as the Spousal Assault Risk Assessment Guide (SARA-V3; Kropp, 2018) as well as the Risk assessment scale of IPH (*Grille d’appréciation du risque d’homicide conjugal*) by Drouin et al. (2012).

2. This tool is used to assess personality disorders and clinical syndromes related to the Diagnostic and Statistical Manual of Mental Disorders (DSM).

3. In the studies reviewed by Kivisto (2015), diagnoses of mental health disorders were established using the DSM and the Millon Multiaxial Clinical Inventory (MCMI) (e.g., Belfrage & Rying, 2004; Liem & Koenraadt, 2008).

4. According to the Canadian Criminal Code, the prescribed sentence for murder is imprisonment for life, with a period of eligibility for parole after 25 years of incarceration for first degree murder and between 10 and 25 years of incarcerations for second degree murder (Ministère de la Justice, 2017).

5. Once the recruitment was completed, the practitioners had no longer access to information about the participants. We thank the practitioners from both organizations for their invaluable collaboration in this study, as well as the participants who contributed to the advancement of knowledge.

6. Other variables were assessed during the interviews, such as impulsivity measures, types of IPV, alcohol and drug abuse. However, these variables were not included in this study.

7. The profile’s names do not include the entirety of the characteristics assessed, but rather represent a summary of the internal dynamic of the men included in the profile. These profiles could be clarified in future studies.

## Author Biographies

**Carolanne Vignola-Lévesque** is a Doctoral Candidate in psychology, research-intervention profile (PhD R/I), at the Department of Psychology, University of Quebec at Trois-Rivières. Her research interests include intrafamilial violence, intimate partner violence, legal psychology, and personality functioning.

**Suzanne Léveillée**, PhD, is a Psychologist and Professor at University of Quebec at Trois-Rivières since 1994. She teaches adult intervention in psychology and psycholegal classes. Her research focuses on the psychosocial issues of intrafamilial violence and deals with the psychosocial and criminological issues of perpetrators of an intrafamilial homicide. She is interested in the psychological changes and the help-seeking of perpetrators of intimate partner violence.
